# Phantom Tumor: A Diagnostic Challenge

**DOI:** 10.7759/cureus.78415

**Published:** 2025-02-03

**Authors:** Rita Vilar da Mota, Sabina Azevedo, Patrícia Sobrosa, Sara Pereira, Ana Rita Oliveira

**Affiliations:** 1 Medicine, Hospital de Viana do Castelo, Unidade Local de Saúde do Alto Minho, Viana do Castelo, PRT; 2 Medicine, Unidade Local de Saúde do Alto Minho, Viana do Castelo, PRT; 3 Internal Medicine, Hospital de Santa Luzia, Unidade Local de Saúde do Alto Minho, Viana do Castelo, PRT; 4 Internal Medicine, Unidade Local de Saúde do Alto Minho, Viana do Castelo, PRT

**Keywords:** hypervolemia, interlobar pleural effusion, lung tumor, phantom tumor, pulmonary congestion, radiological findings, vanishing tumor

## Abstract

The vanishing or phantom tumor of the lung is an uncommon condition that is caused by an interlobar pleural collection of transudative fluid, resulting from pulmonary congestion. The diagnosis is typically made via chest X-ray, where it appears as a nodular opacity, often mistaken for a pulmonary tumor, hence the term "phantom tumor." This condition is most frequently associated with systemic diseases such as heart failure or chronic kidney disease. The lesion generally resolves with the treatment of hypervolemia with diuretic therapy, though recurrence is possible if the underlying disease becomes decompensated.

This report presents two cases of phantom tumors in elderly patients with comorbidities, including heart failure and chronic kidney disease. In both the patients, chest X-rays revealed nodular opacities that resolved after diuretic treatment and fluid management. These cases underscore the importance of early recognition and clinical correlation in distinguishing phantom tumors from true pulmonary nodules, thereby avoiding unnecessary invasive procedures.

To sum up, early detection of phantom tumors enables clinicians to refine differential diagnoses and implement timely therapeutic interventions, as these findings are reversible with appropriate management. This report emphasizes the necessity of integrating careful clinical evaluation with imaging techniques to avoid diagnostic errors and excessive treatments. Thus, raising awareness among physicians and radiologists about this rare but significant condition is vital for the effective management of pulmonary congestion.

## Introduction

The phantom tumor of the lung is a rare radiological finding. It typically manifests as a pleural collection of transudative fluid within the interlobar space, caused by pulmonary congestion stemming from systemic conditions such as heart or kidney failure. On chest X-rays, this fluid collection frequently presents as a nodular opacity, which can be easily mistaken for a pulmonary tumor, sometimes prompting unwarranted diagnostic procedures such as biopsies or surgery. The term "phantom tumor" aptly describes the transient nature of this lesion, as it usually resolves once the underlying condition is appropriately managed.

This phenomenon is most commonly observed in patients with heart failure or chronic kidney disease, both of which contribute to fluid overload and pulmonary congestion. The phantom tumor is most commonly found in men and frequently within the transverse fissure (around three-quarters of the reported cases), but may also occur on the left or near the mediastinum [1-6]. Diagnosing this condition can be challenging, as its appearance on imaging closely resembles that of a neoplasm.

In most cases, the pseudolesion responds well to diuretic therapy aimed at controlling fluid overload, though it may recur if the underlying disease remains untreated. A correct diagnosis is essential to prevent unnecessary or invasive interventions. This report highlights two cases of phantom tumors, emphasizing the importance of distinguishing them from actual pulmonary tumors and the critical role of imaging in confirming the diagnosis.

## Case presentation

Case 1

A 79-year-old male with previous smoking habits and a history of heart failure due to ischemic cardiomyopathy and chronic kidney disease, secondary to renal artery stenosis, presented to the Emergency Department (ED) with symptoms of shortness of breath, orthopnea, and paroxysmal nocturnal dyspnea two days after their onset.

Upon physical examination, the patient appeared comfortable and showed normal vital signs. There was no jugular venous distention or cyanosis. Cardiovascular examination showed a regular rhythm, and respiratory examination revealed clear breath sounds. There was also no peripheral edema.

The clinical presentation was compatible with acute pulmonary edema: the patient was anxious and in moderate respiratory distress, with orthopnea and mild cyanosis around the lips. Vital signs showed elevated blood pressure (170/95 mmHg), tachycardia (110 bpm), and oxygen saturation of 88%, with accessory muscle use. Jugular venous distension was also noticeable. Bilateral basal crackles were heard on lung auscultation, and he also presented bilateral pitting edema in the lower legs, extending to the mid-calf.

**Figure 1:**
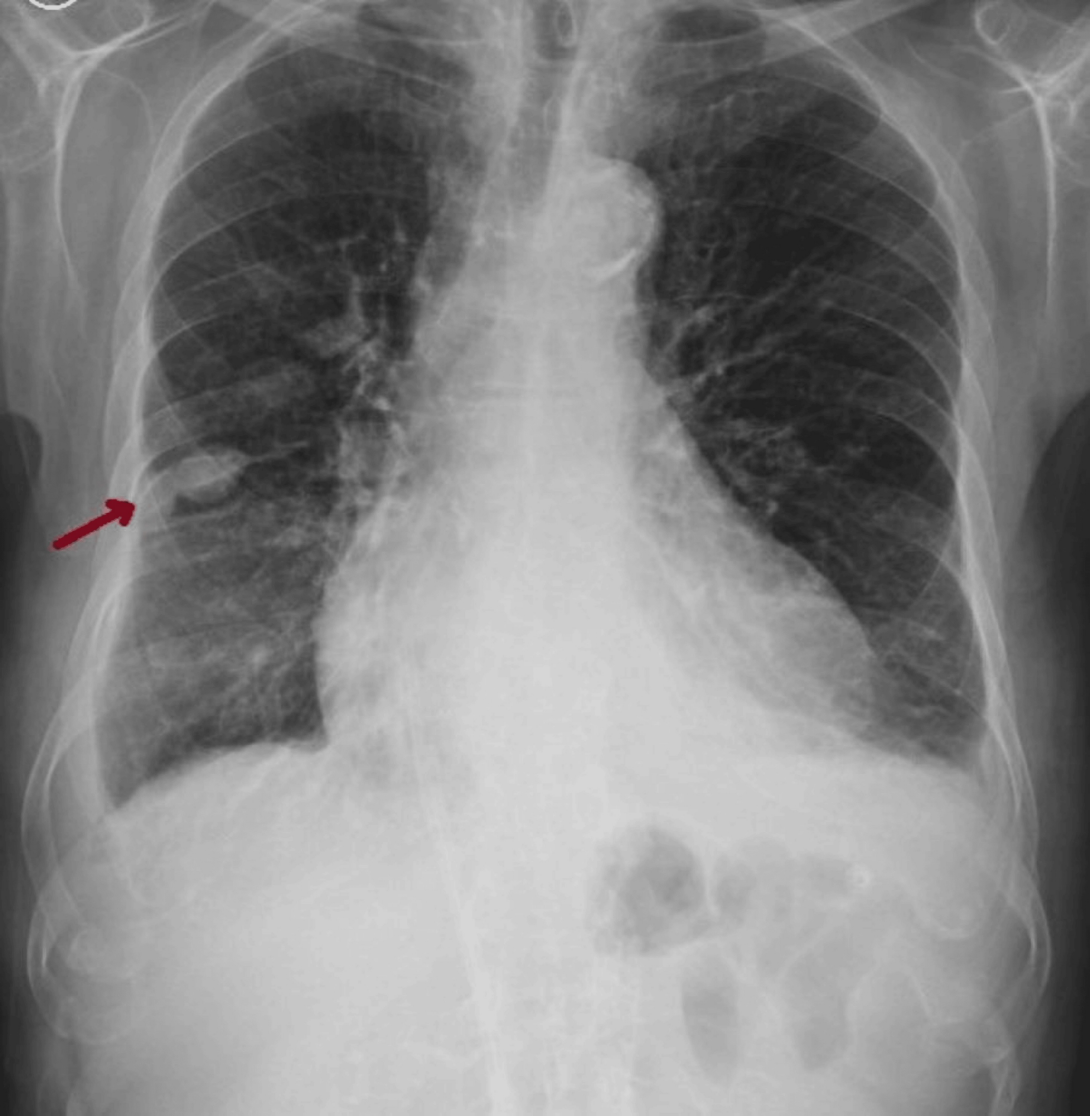
Phantom tumor on chest X-ray

A chest X-ray was performed, revealing a nodular opacity in the right hemithorax (Figure 1). Given the patient's medical history, there was a strong suspicion of a pulmonary tumor, and the lesion was initially concerning for a malignancy. However, due to the patient's underlying conditions and the acute nature of his symptoms, diuretic therapy was initiated to address the acute pulmonary edema.

**Figure 2:**
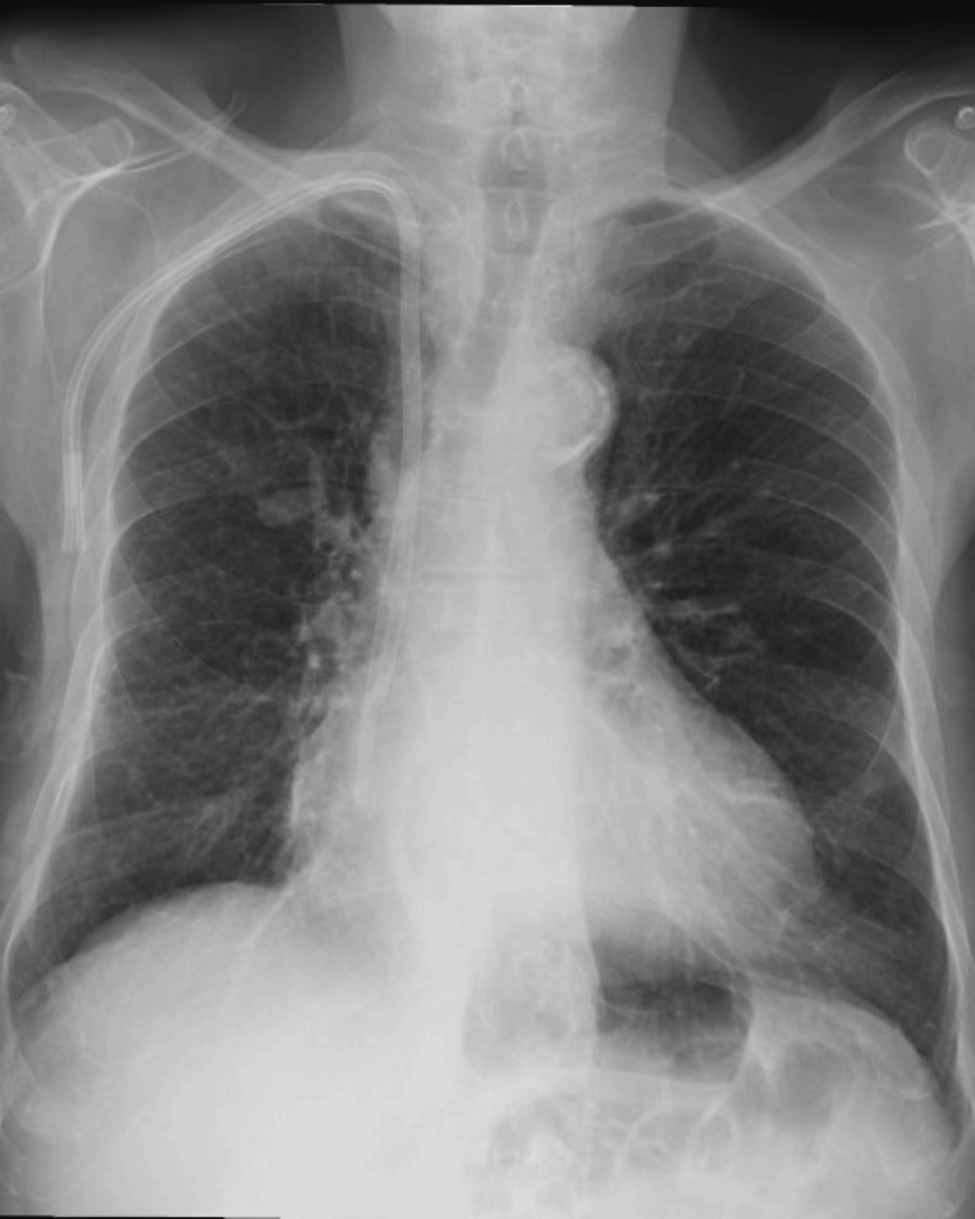
Chest X-ray after diuretic therapy

Following the administration of furosemide, the patient's clinical symptoms improved significantly within 24 hours. A follow-up chest X-ray showed resolution of the previously observed opacity, confirming that the radiological finding was not a true tumor but rather a phantom tumor resulting from pulmonary congestion (Figure 2).

Case 2

**Figure 3:**
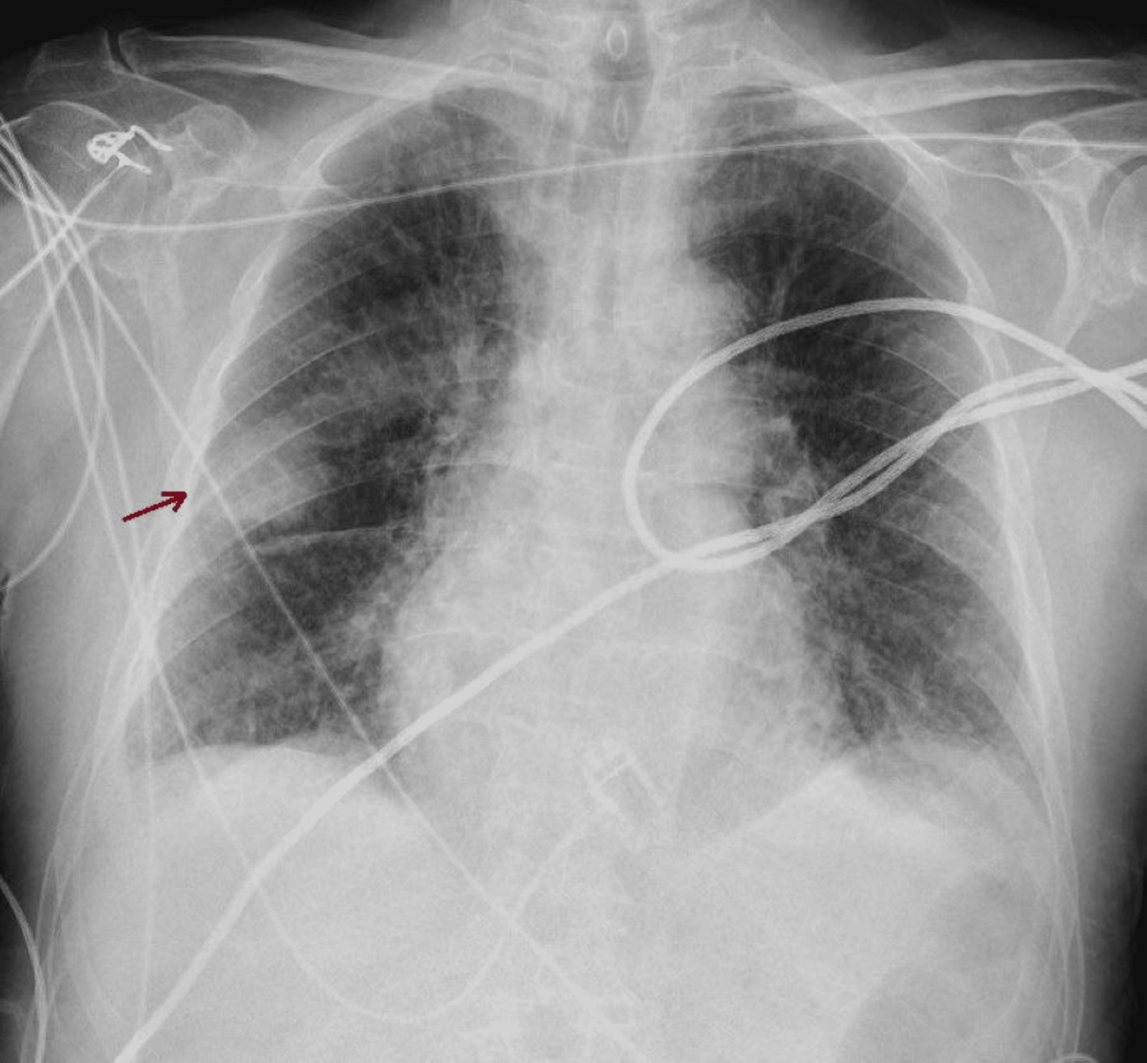
Phantom tumor on chest X-ray

A 94-year-old male with a medical history of hypertension, dyslipidemia, atrial fibrillation, asthma, and chronic kidney disease was admitted to the ED following an ischemic stroke. Although the patient did not exhibit significant respiratory symptoms such as dyspnea, a routine chest X-ray revealed a rounded opacity in the right pulmonary fissure (Figure 3).

**Figure 4:**
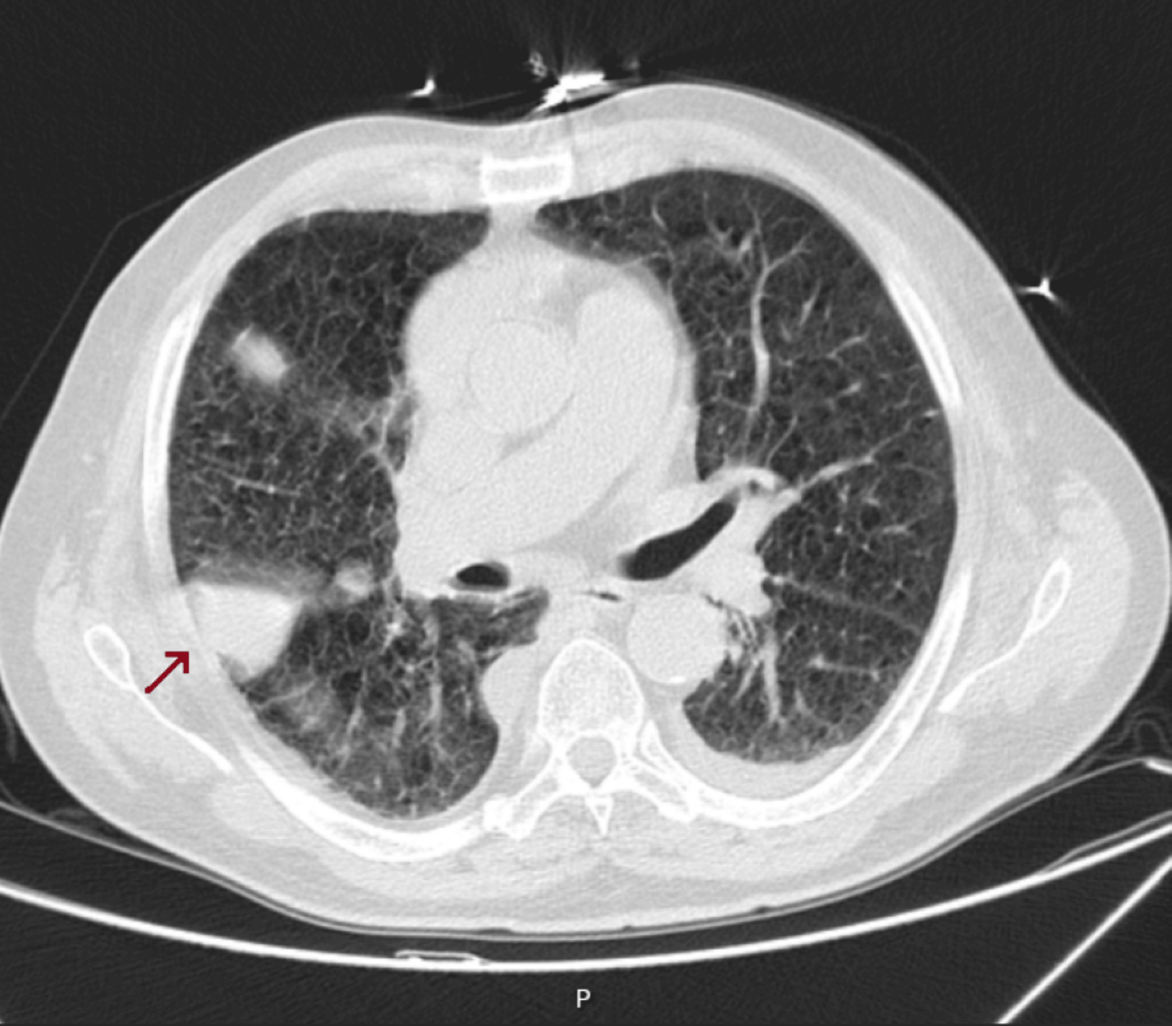
Phantom tumor on CT scan

This finding initially raised concerns about the possibility of a pulmonary tumor, especially given the patient’s advanced age and comorbidities. A chest CT scan was performed to further investigate the visible alteration, which described small volume bilateral pleural effusion, with an elongated nodular image on the right, 6 cm in length, attached to the major fissure (Figure 4).

**Figure 5:**
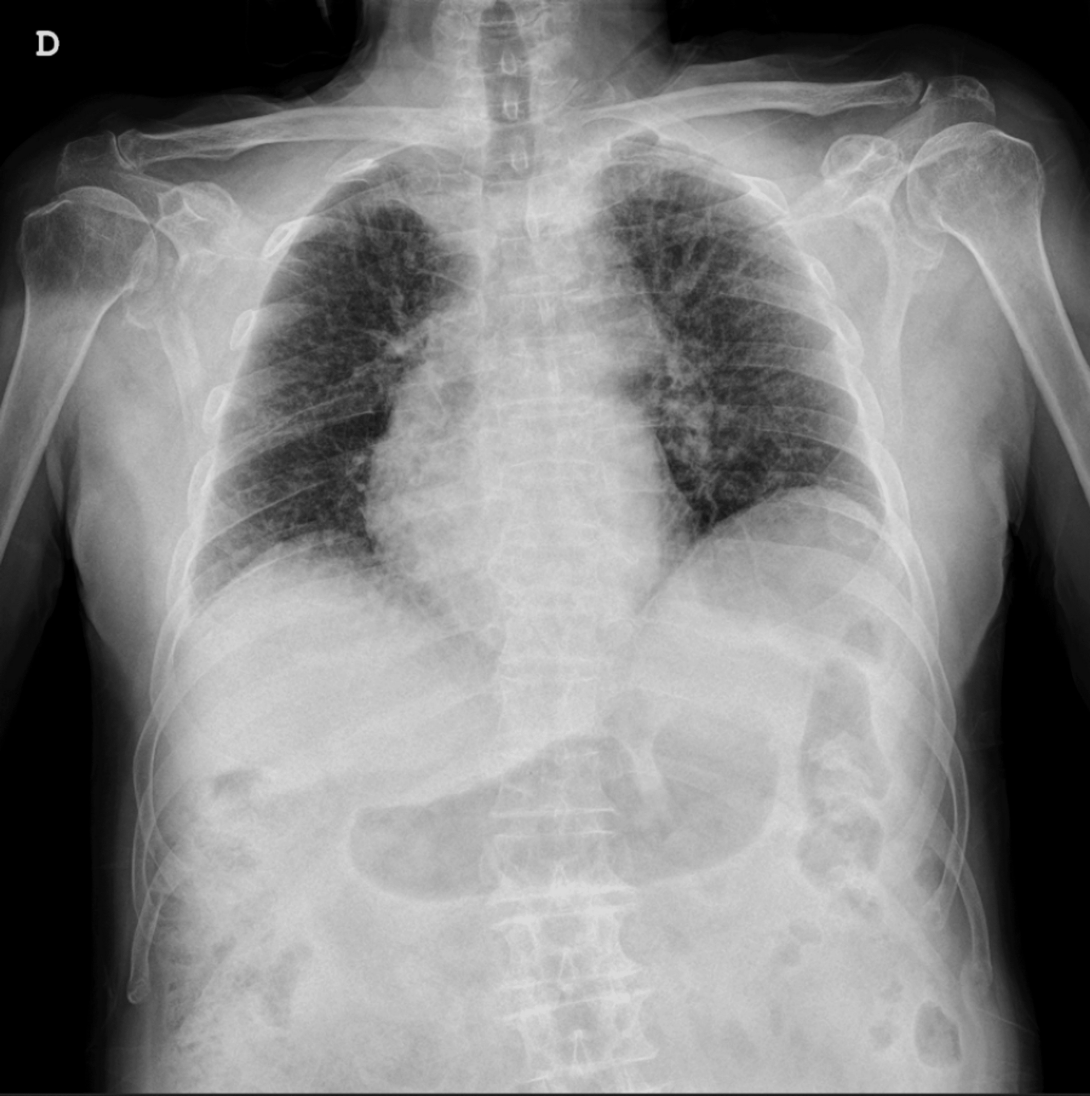
Chest X-ray after diuretic therapy

Given his history of heart and kidney disease, pulmonary congestion was suspected as a potential cause of the radiological finding. Treatment with furosemide was initiated to achieve euvolemia and optimize fluid balance. Within a few days, after achieving euvolemia, a repeat chest X-ray demonstrated the resolution of the opacity, confirming that the finding was consistent with a phantom tumor caused by pleural fluid accumulation rather than a true pulmonary mass (Figure 5).

## Discussion

The phantom tumor of the lung is an uncommon but clinically significant condition that, because of its radiological appearance, may be confused with malignancies or other pathological conditions. It consists of fluid in the interlobar fissures, usually due to pulmonary congestion from cardiac or renal failure. Timely recognition of this condition is critical as such lesions resolve quickly with diuretic treatment and can easily be distinguished from true pulmonary tumors or nodules.

Though the clinical presentation differed in both cases, accurate diagnosis allowed for prompt improvement with conservative treatment, aligning with prior studies, showing similar lesions resolving with diuretic therapy alone [1,3,7-9].

The phantom tumors represent a typical finding of radiological expertise; for this reason, advanced imaging techniques, such as CT, are decisive in ascertaining their fluid nature and excluding possible pathologies. This unusual condition has to be beared in mind by clinicians and radiologists to better recognize the same without overtreatment.

## Conclusions

To sum up, although phantom tumors are rare, it is crucial to consider them as a possible diagnosis in patients showing signs of pulmonary congestion. Confusing a phantom tumor with a more serious condition, such as a pulmonary cancer, can result in unnecessary tests and heightened patient stress. By identifying this benign and reversible condition early, healthcare providers can concentrate on treating the root cause and ensuring appropriate care, namely by avoiding invasive procedures. As such, maintaining a strong level of clinical suspicion and combining findings from physical examinations, imaging studies, and patient history are essential for making an accurate diagnosis and achieving the best outcomes for patients.
